# Punishment-resistant alcohol intake is mediated by the nucleus accumbens shell in female rats

**DOI:** 10.1038/s41386-024-01940-0

**Published:** 2024-07-29

**Authors:** Allison J. McDonald, Panthea Nemat, Thijs van ‘t Hullenaar, Dustin Schetters, Yvar van Mourik, Isis Alonso-Lozares, Taco J. De Vries, Nathan J. Marchant

**Affiliations:** 1 Amsterdam UMC location Vrije Universiteit Amsterdam, Anatomy & Neurosciences, De Boelelaan 1117, 1081 HZ Amsterdam, The Netherlands; 2 Amsterdam Neuroscience, Compulsivity Impulsivity and Attention, Amsterdam, The Netherlands

**Keywords:** Addiction, Striatum, Operant learning, Reward, Motivation

## Abstract

Alcohol use is widespread across many societies. While most people can control their alcohol use, a vulnerable sub-population develops alcohol use disorder, characterized by continued alcohol use despite negative consequences. We used a rat model of alcohol self-administration despite negative consequences to identify brain activity associated with this addiction-like behaviour. We and others have previously shown that response-contingent punishment of alcohol self-administration with mild footshock reliably identifies two sub-populations. One group substantially decreases alcohol self-administration in the face of punishment (punishment-sensitive, controlled) and another group continues alcohol self-administration despite negative consequences (punishment-resistant, addiction-like behaviour). In this study, we aimed to validate this model in females and identify associated brain regions. We trained Long-Evans outbred rats (*n* = 96) to self-administer 20% ethanol, and then introduced response-contingent footshock. We found that female rats consumed more alcohol in unpunished and punished sessions compared to male rats. In one group of rats (*n* = 24, m/f), we identified neuronal activity associated with punishment-resistant alcohol self-administration using the neurobiological marker of activity cFos. We found lower cFos expression in NAcSh associated with punishment-resistant alcohol self-administration. In another group of rats (*n* = 72, m/f), we used chemogenetic inhibition of NAcSh during punished alcohol self-administration. We found that chemogenetic NAcSh inhibition had no effect on unpunished alcohol self-administration but selectively increased punished alcohol self-administration in punishment-resistant female rats. These results indicate that more female rats develop punishment-resistant alcohol consumption, and that NAcSh hypofunction may underlie this phenotype.

## Introduction

Alcohol use disorder (AUD) is characterized by an impaired ability to control alcohol consumption despite negative personal, social, economic or health consequences [1, 2]. Despite widespread alcohol use in society, only a relatively small subpopulation develops AUD [3, 4]. While there is evidence that AUD is a brain disorder [5, 6], the reasons why AUD only develops in a subset of alcohol users are not fully understood.

To investigate underlying neurobiological mechanisms of AUD and other substance use disorders, various rodent behavioural procedures are commonly used [7]. To model use despite negative consequences, strategies include pairing alcohol with bitter-tasting quinine or response-dependent footshock [8–20]. Using footshock punishment, we and others have observed variability, with some rats decreasing alcohol use (punishment-sensitive) and others persisting despite the punishment (punishment-resistance or compulsive use) [10, 12–14, 16, 18]. These models reveal individual differences in alcohol consumption in response to negative consequences, which may reflect the human condition.

The neurobiology of aversion-resistant alcohol use has been the focus of research in many studies [21]. Cortico-striatal circuits are often implicated in clinical studies [22, 23], and deep brain stimulation studies in humans identify the ventral striatum as an effective target for treatment [24, 25]. In rodents, cortico-striatal circuits have also often been associated with punishment-resistant alcohol use, including medial prefrontal cortical (mPFC) projections to nucleus accumbens (NAc) [13, 16], anterior insular (aIC) projections to NAc [16] as well as dopaminergic inputs to the anterior dorsolateral striatum [12, 26]. Subcortical brain regions have also been implicated in punishment-resistant alcohol intake such as mPFC projections to brainstem (peri-aqueductal grey) [18] and PKC-delta neurons in central amygdala [10]. We recently found that neuronal activity (quantified by cFos expression) in NAc Shell (NAcSh), but also paraventricular thalamus and lateral hypothalamus, were associated with alcohol seeking under risk of punishment [27]. Critically, all these studies have exclusively used male rodents, and whether these findings can be extended to females is unknown.

In this study, we aimed to expand the punished alcohol self-administration model to female rats and tested the role of NAcSh in punishment-resistant alcohol intake. We trained male and female outbred Long-Evans rats in punished alcohol self-administration and classified rats as punishment-resistant or punishment-sensitive to characterize these phenotypes. We found that more females became punishment-resistant than males. Using cFos as a marker of neuronal activity [28–30], we found lower NAcSh activity associated with punishment-resistant alcohol intake. Next, we found that chemogenetic inhibition of NAcSh during punished alcohol self-administration in females modulates compulsive-like, but not unpunished, alcohol intake. This work extends the use of translational AUD models to females and provides further evidence that NAcSh plays a role in alcohol use despite negative consequences.

## Materials and methods

### Subjects

Male and female outbred Long Evans rats were used (RjOrl:LE, RGD_151356971, Janvier Labs, France), delivered at 125–149 g (approx. 5–7 weeks old). Rats were same-sex pair-housed on a 12/12-hour reverse light/dark cycle (07:00 lights OFF). All procedures were approved by the Vrije Universiteit Animal Welfare Body and conducted under the authority of the Dutch Central Commission for Animal Research (CCD, permit #AVD1140020187084) in accordance with European law (Directive 2010/63/EU). See Table S1 in Supplementary Materials for more housing details.

### Viral injection surgery for chemogenetic experiments

For NAcSh inhibition, we bilaterally injected hM4Di (AAV-5/2-hSyn1-hM4D(Gi)_mCherry-WPRE-hCHp(A), 6.3 × 10^12^ vg/ml, UZH VVF) or mCherry control (AAV-5/2-hSyn1-chI-mCherry-WPRE-SV40p(A), 6.1 × 10^12^ vg/ml, UZH VVF) viral vectors in NAcSh (AP + 1.7, ML ± 2.4, DV-7.8, 10°; 0.5 uL per hemisphere). See Supplementary Materials for details.

### Apparatus

Rats were trained to self-administer alcohol in standard operant chambers inside ventilated sound-attenuated cabinets (Med Associates Inc., USA). Each chamber consisted of left (active) and right (inactive) retractable levers on either side of the magazine, connected to a syringe pump which dispenses 0.12 mL 20% (v/v) ethanol per delivery. A white cue light (40 mA) above the active lever and a speaker were installed for audiovisual cues. The metal grid floors were connected to aversive stimulator/scrambler modules to deliver footshocks.

### Behavioural procedure

#### Home-cage intermittent alcohol access two-bottle choice procedure

One week after arrival, rats began the intermittent alcohol access two-bottle choice procedure [31, 32], similar to procedures in our previous experiments [27, 33, 34]. Rats received 24-h alcohol access (3 x per week, 20% alcohol) then 24–48 h without access for 4 weeks, for a total of 12 × 24 h sessions. See Supplementary Materials for details. Where relevant, surgeries took place after home-cage alcohol access.

#### Punished alcohol self-administration training procedure

30-minute alcohol self-administration sessions took place during the dark phase, 5–6 days per week. There were 4 training phases (Magazine training, Alcohol self-administration, Progressive ratio test, and Punished alcohol self-administration). Final alcohol self-administration sessions were run at a fixed ratio schedule of 3 (FR3), where 3 active lever presses were required for alcohol reward (0.12 mL, 20%). Punished sessions were identical, except with a 33% probability of footshock upon the second active lever press in the FR3 sequence. Footshock intensity was increased after 3–5 session (0.20 mA, 0.25 mA, 0.30 mA). See Supplementary Materials for details.

##### Behavioural exclusion criteria

Rats were excluded from analysis if they completed < 9 active lever presses (<3 alcohol deliveries) in the alcohol self-administration baseline period.

##### Suppression ratio

We calculated a Suppression Ratio (SR) [14, 35] to identify the effect of punishment on alcohol self-administration. SR provides a measure of responding in punishment as a function of pre-punishment self-administration rates and was calculated using the following formula: *SR = Punished / (Baseline + Punished)*. Baseline alcohol self-administration rates are average active lever presses from the final 3 self-administration sessions.

##### Punishment-resistance score

We made a Punishment-Resistant score to classify rats as punishment-resistant or punishment-sensitive, using the average SR from the last two punished sessions at 0.25 mA. At this shock intensity, we saw individual differences in punished alcohol self-administration emerge [14] and we reliably observed a similar behavioural response (startle jump) across all individuals (Supplementary Materials, Fig. S3). For analysis, we preserved the punishment-resistance score as a continuous variable wherever possible, to avoid having to create an arbitrary threshold [36]. When analyses required a categorical variable, the top third of the punishment-resistant score distribution were classified as punishment-resistant and the bottom third punishment-sensitive.

### Chemogenetic test procedure

For chemogenetic experiments, rats were randomly allocated to receive viral vectors encoding either hM4Di or mCherry, within blocking factors of Sex, Surgeon, and Surgery Day. Rats were first habituated to injections (NaCl, 0.9%, i.p., 1 mL/kg) at least twice prior to any chemogenetic test sessions. We used a repeated-measures design, where each rat received each ligand/dose, counterbalanced across days.

We prepared deschloroclozapine (DCZ) (MedChem Express, USA), a potent chemogenetic ligand [37], first by dissolving in glacial acetic acid (0.1% final DCZ volume), and then diluting in sterile saline (NaCl, 0.9%) to a final concentration of 0.1 mg/mL (pH 7.3). DCZ was stored at -20 °C and thawed to room temperature on test days. Injection volumes were 1 ml/kg, and the dose was 0.1 mg/kg.

### Perfusion, brain sectioning, staining & mounting

We used standard procedures similar to our past work [27, 33, 38–41]. For cFos immunohistochemical staining, we used rabbit anti cFos primary antibody (1:2000), Phospho-c-Fos (Ser32(D82C12)) with avidin-biotin complex DAB (diaminobenzidine) technique (Vectastain Elite ABC-HRP kit with peroxidase, Vector Laboratories). cFos-positive nuclei were automatically counted using the analyze particles function in ImageJ (v. 1.53c). For chemogenetic experiments, tissue was DAPI-stained, and slides were scanned at ×20 magnification to image DAPI-stained nuclei (DAPI filter) and mCherry expression (Texas Red filter) (Vectra Polaris slide scanner, Akoya Biosciences). See Supplementary Materials for details.

### Experimental design

#### Experiment 1. Behavioural characterization of punished alcohol self-administration in male and female rats

For this analysis, we combined all cohorts which went through comparable behavioural training regardless of viral expression. In total, 72 rats (36 male, 36 female) were included. We compared behaviour in punished alcohol self-administration between Sexes and Punishment-Resistance Score.

#### Experiment 2. Neuronal activity associated with punished alcohol self-administration

Twenty-four rats (12 male, 12 female) went through the behavioural procedure described above. After punished alcohol self-administration training, rats completed a final punished (*n* = 18) or unpunished (*n* = 6) alcohol self-administration test session. Rats were classified by their Punishment-Resistance Score, and those with the highest and lowest 9 values were chosen to undergo the punished alcohol self-administration test. Test sessions were identical to training sessions and responses were reinforced with alcohol and/or footshocks. Rats were perfused 60 min after the start of the behavioural test.

#### Experiment 3. Novel chemogenetic ligand deschloroclozapine behavioural validation

We ran a behavioural validation experiment to test the novel chemogenetic ligand deschloroclozapine (DCZ) in rats [37]. Rats (*n* = 8) were injected with hM4Di or mCherry viral vectors in the VTA/SNc and later completed open field locomotor tests after saline (SAL), 0.05 mg/kg DCZ, and 0.10 mg/kg DCZ injection on separate days [37, 42]. Total distance travelled (cm) was calculated from recorded videos using ezTrack [43]. See Supplementary Materials for details.

#### Experiment 4. NAcSh chemogenetic inhibition during punished alcohol self-administration

Seventy-two rats (36 male, 36 female) underwent viral injection surgery and then punished alcohol self-administration training. Six rats were excluded due to health concerns, 6 rats were excluded based on not reaching behavioural criteria, and 14 rats were excluded because they did not show bilateral virus expression in NAcSh. The final analysis consisted of 22 rats expressing hM4Di (11 male, 11 female) and 24 rats expressing mCherry (10 male, 14 female).

Chemogenetic tests were performed at the end of: Unpunished (FR3), Progressive Ratio, 0.25 mA Punishment and 0.30 mA Punishment self-administration training. Test sessions were identical to training sessions in each behavioural phase. Rats were tested in a repeated-measures counter-balanced design, injections (Saline or DCZ) were 5 min prior to session start.

### Statistical analyses

Data was analyzed and visualized using R Software [44]. Values of *p* < 0.05 were considered statistically significant. Non-parametric alternatives were used if statistical assumptions were not approximately met. Significant effects were followed up with Tukey or Bonferroni-Holm post-hoc tests. In Experiment 1, we used ANOVAs to analyze behavioural variables across Session (within-subject factor) and by Sex or Punishment-Resistance (between-subject factors). In Experiment 2, we used ANOVAs and Spearman’s correlations to analyze cFos counts in each brain region by Test Groups (between-subject factor; Unpunished, Punishment-Sensitive, Punishment-Resistant). In Experiment 3, we performed separate analyses for males and females based on the population differences we observed between sexes in punishment-resistant alcohol intake. We used separate generalized linear mixed models (GLMM) to identify Virus x Ligand x Punishment-Resistance interaction effects on test behaviour in each sex. Using GLMMs allowed us to preserve Punishment-Resistance Score as a continuous variable and avoid making an arbitrary threshold, as well as account for non-normal data. Data are presented as mean ± SEM, unless otherwise specified. Visualizations include individual datapoints and identify sex wherever possible. See Supplementary Materials and results for additional information.

## Results

### Experiment 1. Behavioural characterization of punished alcohol self-administration in male and female rats

#### More female rats become punishment-resistant after higher alcohol self-administration

**Fig. 1:**
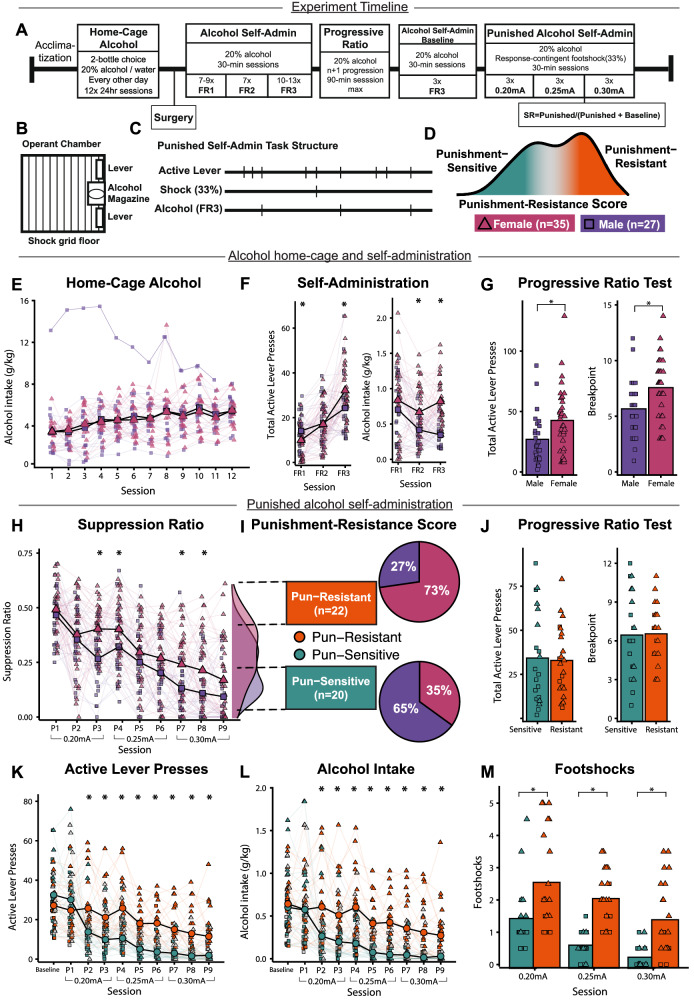
Behavioural characterization & sex differences in punished alcohol self-administration. **A** Behavioural procedure. **B** Operant chamber setup. **C** Punishment procedure. **D** Suppression Ratio (SR = Punished responding / (Baseline + Punished responding)). Average SR at 0.25 mA was used to create a Punishment-Resistance Score which we used to classify rats as punishment-resistant (high SR, orange) or punishment-sensitive (low SR, green). **E–H** Alcohol intake across punished alcohol self-administration phases in males (*n* = 27, purple squares) and females (*n* = 36, pink triangles). *Difference between Sexes, *p* < 0.05. **E** Alcohol intake (g/kg) during home-cage alcohol access sessions. **F** Final average total active lever presses (left) and alcohol intake (g/kg) (right) during 30-min unpunished alcohol self-administration sessions. **G** Total active lever presses (left) and breakpoint (right) during progressive ratio test. **H** SR across punished alcohol self-administration sessions. **I** (Left) Density histograms of average 0.25 mA Suppression Ratios (SR) in males (purple) and females (pink). Highest 33% SR are punishment-resistant (*n* = 22, orange), lowest 33% are punishment-sensitive (*n* = 20, green). The punishment-resistant group was 73% female (pink), and the punishment-sensitive group was 65% male (purple). **J–M** Punished alcohol-self administration data classified as punishment-resistant versus punishment-sensitive (Males = squares, females = triangles). *Difference between SR categories, *p* < 0.05. **J** Progressive ratio test. **K** Total active lever presses for final FR3 average (Baseline) and every punished session. **L** Alcohol intake (g/kg) from unpunished Baseline and every punishment session. **M** Average footshocks received at 0.20 mA, 0.25 mA and 0.30 mA.

Rats were trained in the punished self-administration procedure (Fig. 1A–D). We found that male and female rats show similar increased alcohol intake (g/kg) across sessions in the home-cage (Fig. 1E), but males displayed higher alcohol preference by the final sessions (Fig. S1). However, by the end of alcohol self-administration training, female rats performed more alcohol lever presses, consumed more alcohol, and showed a higher motivation for alcohol than male rats (Fig. 1F, G). After the first few punished alcohol sessions, females developed higher punishment-resistance scores than males (Fig. 1H, Table S2). Importantly, punishment-resistance is stable across the tested punishment intensities (Fig. S2), and males and females do not show differences in non-contingent shock sensitivity (Fig. S3). See Supplementary Materials for details.

#### Punishment-resistance emerges without prior differences in unpunished alcohol intake

See Fig. 1A, C for punished alcohol self-administration procedure details. Consistent with past work, at medium shock intensities, some rats continued alcohol responding despite footshock (high SR, Punishment-Resistant, *n* = 22), while others greatly decrease their responding (low SR, Punishment-Sensitive, *n* = 20) (Fig. 1D, H, I). More females and fewer males than expected were classified as Punishment-Resistant (*X*^*2*^(2) = 6.217, *p* = 0.045) (Fig. 1I, Table S2). Below, we examined alcohol self-administration between punishment-resistant and -sensitive rats both prior to and during punishment to better characterize their phenotypes.

##### Progressive ratio test

We found no differences in total active lever presses (*t(*40) = 0.19, *p* = .851) or breakpoint (*t*(40) = –0.11, *p* = 0.912) in the progressive ratio test between subsequently classified punishment-resistant and -sensitive rats (Fig. 1J).

##### Active lever pressing and alcohol intake

We found a significant Punishment-Resistance Score x Session interaction (*F*(5.19,207.42) = 9.27, *p* < 0.001, η^2^_G_ = 0.11) for active lever pressing across baseline and punished sessions (P1–P9) (Fig. 1K). While punishment-sensitive and -resistant rats showed similar active lever presses at unpunished baseline (*p* = 0.21) and the first punished session (P1, *p* = 0.23), punishment-resistant rats pressed more during all other punished sessions (P2: *p* = 0.007; P3: *p* = 0.003; P4–P8: *ps* < 0.001; P9: *p* = 0.003), not only in the sessions in which they were classified (P5-P6, 0.25 mA). Both punishment-resistant and -sensitive rats showed a significant effect of Session (*ps* < 0.001). While punishment-resistant rats only significantly decreased pressing from the first to the second 0.25 mA session (P4-P5, *p* = 0.012), punishment-sensitive rats significantly decreased lever pressing from the first to the second session at 0.20 mA (P1-P2, *p* = 0.005) and 0.25 mA (P4-P5, *p* = 0.027). Similarly, we found a significant Punishment-Resistance Score x Session interaction (*F*(4.53,181.18) = 4.97, *p* < 0.001, η^2^_G_ = 0.05) when accounting for alcohol intake in g/kg (Fig. 1L). See Supplementary Materials for details.

##### Footshocks received

We found no significant Punishment-Resistance Score x Session interaction (*F*(1.63,65.06) = 0.75, *p* = 0.450) for footshocks received (expressed as the final 2 day average at each intensity), but there were main effects of Punishment-Resistance Score (*F*(1,40) = 25.31, *p* < 0.001, η^2^_G_ = 0.30) and Session (*F*(1.63,65.06) = 33.59, *p* < 0.001, η^2^_G_ = 0.21) (Fig. 1M). Punishment-resistant rats received more shocks than punishment-sensitive rats at every intensity, not only at 0.25 mA from which they were classified (0.20 mA: *p* = 0.006, 0.25–0.30 mA: *ps* < 0.001). Both punishment-resistant (0.20 mA–0.25 mA: *p* = 0.036; 0.25 mA–0.30 mA: *p* = 0.002) and -sensitive rats (0.20 mA–0.25 mA: *p* = 0.002; 0.25 mA–0.30 mA: *p* = 0.004) received fewer shocks as shock intensity increased. Importantly, punishment-resistant and -sensitive rats showed no differences in shock sensitivity in a non-contingent shock test (Fig. S3).

Overall, these data indicate that punishment-resistant rats do not differ from punishment-sensitive rats in baseline alcohol self-administration or motivation for alcohol prior to punishment. After the first punishment session, however, punishment-resistant rats show higher alcohol self-administration rates and receive more foot shocks than punishment-sensitive rats. Differences between punishment-resistant and -sensitive groups were observed in all punishment sessions, not only in sessions used for classification. And notably, more females become punishment-resistant than males.

### Experiment 2. Neuronal activity associated with punished alcohol self-administration

**Fig. 2:**
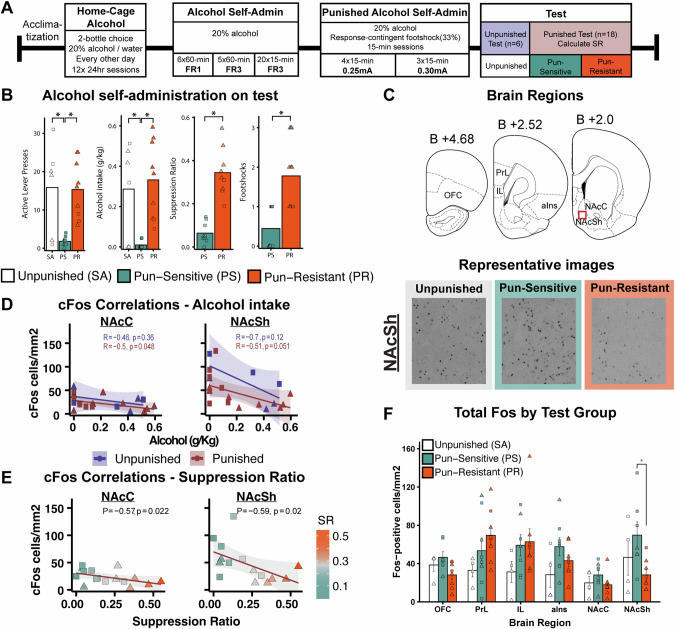
Neuronal activity associated with punished alcohol self-administration. **A** Behavioural Procedure. The test consisted of unpunished (*n* = 6 (3 m, 3 f), white), punishment-sensitive (*n* = 9 (7 m, 2 f), green) and punishment-resistant groups (*n* = 9 (2 m, 7 f), orange). **B** Alcohol self-administration during test session (male = square, female = triangle). Suppression ratio (SR) and footshocks are also shown for punishment-sensitive (green) and punishment-resistant (orange) groups. *Difference between Test Groups, *p* < 0.05. m, Male; f, Female. **C** Representative illustration of analyzed brain regions (top). Representative images of cFos-positive cells in a sub-region of the analysed NAcSh region of interest (bottom). **D** Significant correlations between total active lever presses and cFos (male = square, female = triangle). **E** Significant correlations between SR and cFos in punished alcohol self-administration (Male = square, Female = triangle). **F** cFos-positive cells/mm^2^ (mean ± SEM) in analyzed brain regions by Test Group. *Difference between Test Groups, *p* < 0.05. OFC orbitofrontal cortex, PrL prelimbic cortex, IL infralimbic cortex, aIns anterior insula, NAcC nucleus accumbens core, NAcSh nucleus accumbens shell.

Our next aim was to compare neuronal activity between punishment-resistant, punishment-sensitive, and unpunished alcohol self-administration, assessed through cFos, an indirect marker of neuronal activity. After punished alcohol self-administration, rats completed punished (*n* = 18 (9 m, 9 f)) or unpunished (*n* = 6 (3 m, 3 f)) final test sessions (Fig. 2A). Figure 2B shows behavioural measures of punishment-resistant (*n* = 9 (2 m, 7 f)), punishment-sensitive (*n* = 9 (7 m, 2 f)) and unpunished (*n* = 6 (3 m, 3 f)) groups during the cFos test. Based on small group sizes due to unexpected population sex differences (too few punishment-resistant males), we could not add Sex as a variable in cFos analyses. See Supplementary Materials for details. Brain regions analyzed for cFos and representative images of cFos-positive cells across Test Groups are shown in Fig. 2C.

#### Punishment-resistance is associated with lower NAcSh cFos cell counts

We first tested correlations between total cFos counts and total active lever presses in unpunished and punished alcohol self-administration separately. We found no significant correlations in OFC, PrL, IL or aIC in either unpunished or punished test conditions (Table S3). We did find significant negative correlations in both NAcC (*r*_*S*_ = -0.60, *p* = .014) and NAcSh (*r*_*S*_ = –0.60, *p* = 0.018) in the punished test condition, but not the unpunished test condition (Fig. 2D). We found similar results when accounting for alcohol intake in g/kg, where higher intake under punishment negatively correlated with cFos-positive cells in NAcC (*r*_*S*_ = –0.55, *p* = 0.027) and NAcSh (*r*_*S*_ = –0.63, *p* = 0.012) (Table S3).

Next, we examined correlations between total cFos-positive cells and punishment-resistance score (suppression ratio) in the punished test condition (Fig. 2E). We found no significant correlations in any cortical brain region tested (OFC, PrL, IL, aIC) (Table S3), but did find significant negative correlations in NAcC (*r*_*S*_ = –0.57, *p* = 0.022) and NAcSh (*r*_*S*_ = -0.59, *p* = .020), such that punishment-resistant rats (high SR) showed lower NAcSh activity.

Finally, we tested for overall test group differences in cFos expression across analyzed brain regions (Fig. 2F). We identified a significant effect of Test Group in NAcSh (*F*(2,16) = 3.71, *p* = 0.047, *η*^*2*^_*G*_ = 0.32), but no other brain regions analyzed (OFC, PrL, IL, aIC, NAcC, *ps* > .05). In NAcSh, punishment-resistant rats had significantly lower total cFos counts than punishment-sensitive rats (*p* = 0.038).

### Experiment 3. Novel chemogenetic ligand deschloroclozapine behavioural validation

We determined that 0.10 mg/kg DCZ was an effective dose for chemogenetic inhibition in rats. See Supplementary Materials and Fig. S4 for details.

### Experiment 4. NAcSh chemogenetic inhibition during punished alcohol self-administration

**Fig. 3:**
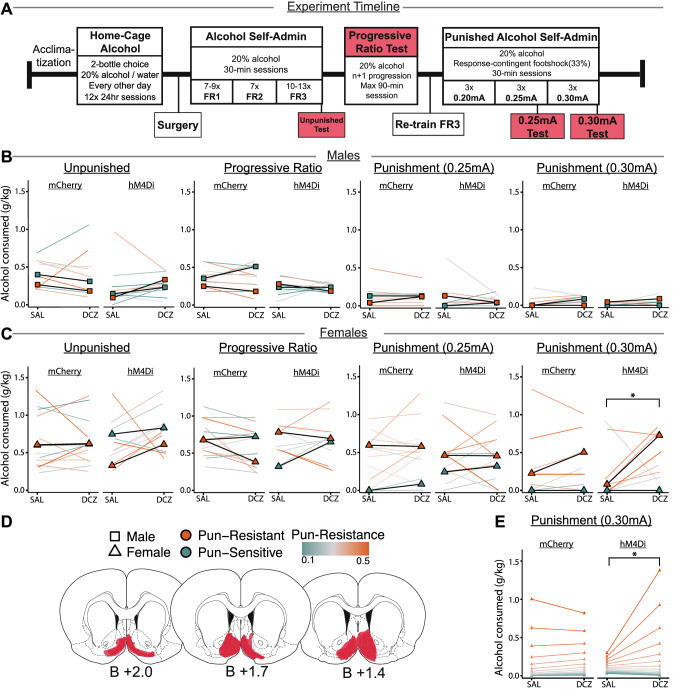
Chemogenetic inhibition of NAcSh during punished alcohol self-administration. **A** Experimental timeline. Effect of NAcSh inhibition on alcohol intake (g/kg) in (**B**) males (mCherry: *n* = 10, hM4Di: *n* = 11) and females (**C**, **E**) (mCherry: *n* = 14, hM4Di: *n* = 11) during alcohol self-administration, progressive ratio test, punished alcohol self-administration at 0.25 mA and punished alcohol self-administration at 0.30 mA. Data expressed as Punishment-Resistance Score (Punishment-sensitive = green, punishment-resistant = orange) medians and individual datapoints. Individual datapoints are coloured using the continuous variable of Punishment-Resistance Score (Low = green, mid = grey, high = orange) and sex is indicated by squares (males) and triangles (females). Separate graphs in each panel show mCherry (left) or hM4Di rats (right) and each graph compares the repeated measure factor Ligand (Saline (SAL) vs. Deschloroclozapine (DCZ)). *Virus-hM4Di x Ligand-DCZ x Punishment-Resistance Score interaction effect, *p* < 0.05. **D** Bilateral expression of hM4Di in NAcSh. **E** Significant model predictions from synthetic data to illustrate significant Virus-hM4Di x Ligand-DCZ x Punishment-Resistance Score interaction effect in females at 0.30 mA, *p* < 0.05.

Experiment 2 showed that greater punishment-imposed suppression of alcohol self-administration is associated with higher NAcSh activity. Therefore, in the next experiment we aimed to test whether chemogenetic inhibition of NAcSh would increase punished alcohol intake. We trained rats after NAcSh injections of viral vectors encoding the inhibitory chemogenetic receptor hM4Di or control mCherry (Fig. 3A, D). Based on population sex differences in punished alcohol self-administration, we performed separate analyses for males (hM4Di: *n* = 11, mCherry: *n* = 10) and females (hM4Di: *n* = 11, mCherry: *n* = 14) [45, 46]. Alcohol intake (g/kg) for all chemogenetic tests are shown in Fig. 3B for males and Fig. 3C, E for females. We focus on alcohol intake (g/kg) to account for the weight differences between males and females. See Supplementary Materials for additional results (Table S4, Fig. S5).

#### NAcSh inhibition does not affect unpunished alcohol self-administration

In the unpunished alcohol self-administration test, (Fig. 3B, C), we found no Virus-hM4Di x Ligand-DCZ x Punishment-Resistance Score interaction in males (*β* = –0.47, SE = 2.30, *Z*(42) = –0.20, *p* = 0.839) or females (*β* = 0.33, SE = 2.01, *Z*(50) = 0.16, *p* = 0.870), nor a Virus-hM4Di x Ligand-DCZ interaction in males (*β* = 0.21, SE = 0.61, *Z*(42) = 0.34, *p* = 0.732) or in females (*β* = 0.33, SE = 0.65, *Z*(50) = 0.51, *p* = 0.614). Similarly, for alcohol intake in the progressive ratio test, we found no Virus-hM4Di x Ligand-DCZ x Punishment-Resistance Score in males (*β* = 0.36, SE = 1.65, *Z*(42) = 0.22, *p* = 0.828) or in females (*β* = –1.33, SE = 1.24, *Z*(50) = –1.07, *p* = 0.286), nor a Virus-hM4Di x Ligand-DCZ effect in males (*β* = –0.10, SE = 0.43, *Z*(42) = –0.24, *p* = 0.809) or in females (*β* = 0.64, SE = 0.42, *Z*(50) = 1.53, *p* = 0.126). Thus, inhibition of NAcSh has no effects on either unpunished alcohol self-administration or motivation for alcohol in the progressive ratio test, in either sex. For both alcohol self-administration and progressive ratio, these data also show that subsequent punishment-resistance has no relationship to the lack of effect of NAcSh inhibition.

#### NAcSh inhibition has no effect on punished alcohol intake at lower shock intensities

In the 0.25 mA punished alcohol self-administration test (Fig. 3B, C), we found no Virus-hM4Di x Ligand-DCZ x Punishment-Resistance Score interaction for alcohol intake in males (*β* = –8.40, SE = 4.99, *Z*(42) = –1.68, *p* = 0.093) or females (*β* = –4.50, SE = 3.14, *Z*(50) = –1.44, *p* = 0.151) nor a Virus-hM4Di x Ligand-DCZ interaction in males (*β* = 1.96, SE = 1.39, *Z*(42) = 1.40, *p* = 0.160) or females (*β* = 1.37, SE = 1.15, *Z*(50) = 1.23, *p* = 0.221). Thus, we found that NAcSh inhibition has no effect on punished alcohol intake at lower shock intensities in either sex, regardless of punishment-resistance score.

#### NAcSh inhibition increases alcohol intake exclusively in punishment-resistant female rats at higher shock intensities

In the 0.30 mA punished self-administration test (Fig. 3B, C), we found a Virus-hM4Di x Ligand-DCZ x Punishment-Resistance Score interaction in females (*β* = 10.79, SE = 5.37, *Z*(50) = 2.01, *p* = 0.045) but not males (*β* = –5.52, SE = 9.08, *Z*(367) = –0.61, *p* = 0.543) for alcohol intake (g/kg). Using synthetic data, we showed that in females expressing hM4Di, the model predicted differential effects of the DREADDs ligand DCZ depending on Punishment-Resistance Score, in which higher punishment-resistance scores predicted strongly increased alcohol intake upon NAcSh inhibition and lower scores showed no effects (Fig. 3E). Overall, these results indicate a relationship between Punishment-Resistance Score and the effect of NAcSh inhibition on punished alcohol use, such that inhibition exacerbates the punishment-resistance phenotype exclusively in females.

## Discussion

In this study, we extended the punished alcohol self-administration behavioural procedure to female rats. We found that more female rats became punishment-resistant compared to male rats, and that females show higher motivation and alcohol self-administration at the end of training. Using cFos as a marker of neuronal activity, we showed that higher suppression of alcohol intake by punishment was associated with higher NAcSh activity. Finally, using chemogenetics, we found that NAcSh inhibition led to exacerbation of the punishment-resistance phenotype in female rats.

### Behavioural phenotype of punishment-resistant alcohol intake

Here we demonstrate that outbred Long-Evans male and female rats also show individual variability in their response to punishment of alcohol self-administration, which is a comparable phenomenon observed previously in male Alcohol Preferring P rats [14]. Recently, other studies have identified similar individual variability in response to punishment using other outbred strains of rat [10, 12, 16]. Indeed, a comparable observation can be identified in mice responding for intra-cranial self-stimulation of dopamine neurons [47, 48], rats lever pressing for food [49], and humans in a punishment task with comparable parameters [50].

Comparison of alcohol self-administration between the punishment-resistant and punishment-sensitive groups revealed no differences in prior alcohol intake levels and no differences in motivation for alcohol. Similar evidence is found in studies testing aversive-resistant alcohol consumption (e.g. with footshock or quinine adulteration), showing that alcohol intake and alcohol intake despite negative consequences are uncoupled [10, 12–14, 16]. These observations suggest that motivation for alcohol, as measured by total alcohol intake or progressive ratio, and resistance to punishment are mediated by orthogonal constructs. An important implication of this is that each construct may separately contribute to addiction-related phenomena.

An alternative possibility describing the punishment-resistance phenomenon is that the punishment-resistant rats have lower physical sensitivity to the shock. To address this, we tested shock sensitivity threshold and found that this did not predict punishment resistance. Thus, we show that punishment-resistant subjects are equally sensitive to the footshock sensation tested in a non-contingent manner. Similar results have been reported in other studies that observed individual differences using footshock as punishment [10, 51].

If punishment-resistance does not emerge from differences in prior alcohol consumption, motivation for alcohol, or sensitivity to shock, the question remains why some individuals develop persistent alcohol use despite negative consequences. A few alternative behavioural hypotheses could be aberrant punishment learning or decreased Pavlovian fear response, although they have not been tested here. Jean-Richard-dit-Bressel et al. (2019) tested these ideas on rats trained to lever press for food pellets and found no differences in Pavlovian fear responses between punishment-sensitive and resistant groups of rats [49]. This indicates that punishment-resistant rats may have a deficit in formation of operant punishment associations.

### Preserving punishment-resistant score as a continuous variable to analyze individual differences

Different phenotypic classification methodologies have been used to explore individual variation to (punished) drug-seeking behaviour [10, 12–14, 16, 18, 52], which can make it difficult to compare across studies. When investigating individual differences, the methodology used to categorize individuals into subgroups based on a continuous variable has important consequences on data interpretation. Here we used two approaches. To characterize behavioural phenotypes in a large sample, we split the continuous variable, Punishment-Resistance Score, into two categories (punishment-resistant and punishment-sensitive) based on our observation of potential bimodal distribution at shock intensity 0.25 mA. Bimodal subpopulations have previously been observed from bimodal histograms, and the middle trough can be used to mark a threshold [10, 14]. However, this approach can be underpowered in smaller sample sizes, and creating a threshold to binarize a continuous variable relies on an assumption that individuals on either side of the threshold are categorically different, which in this case, is difficult to justify [36, 53]. For this reason, we analysed the neurobiological data with Punishment-Resistance Score preserved as a continuous variable. We analysed cFos data using correlations, and chemogenetic NAcSh inhibition using generalized linear mixed models [54, 55], which allowed for the preservation of continuous variables and accounted for non-normal data distributions. This approach allowed us to identify complex effects of NAcSh inhibition on punishment-resistant alcohol use while avoiding interpretational issues related to creating an arbitrary threshold to form behavioural categories.

### Sex differences in alcohol intake despite negative consequences

We found that more female rats became punishment-resistant compared to male rats, and that females show higher motivation and alcohol self-administration at the end of training. Many studies identifying neural mechanisms underlying punishment-resistant alcohol use have not included female rodents [10, 12–14, 16, 18, 27]. A recent study demonstrated a comparable effect in Wistar rats, showing that female rats have higher levels of punished alcohol self-administration compared to males [56]. Recently, Sneddon et al. used female mice in a punished alcohol self-administration procedure, and also found that female mice persist with punished alcohol use at higher shock intensities than male mice [57]. Another study using quinine-adulterated alcohol also found a greater preponderance of females in the aversion-resistant group [58, 59], suggesting that this phenomenon generalizes across different punishers.

In this study, we did not directly test the effects of the oestrous cycle on unpunished or punished alcohol intake. An important role for oestrous cycle in incubation of cocaine craving has been identified [60], illustrating that fluctuations in hormones can directly impact drug seeking. Previous studies in alcohol self-administration and punishment have found no relationship between these and the oestrous cycle [56, 61, 62].

In humans, AUD rates are increasing faster in women than in men, narrowing the gender gap, and further exaggerating the importance of including females in pre-clinical research for future treatment development [63, 64]. By including females in our study, we aid in developing an understanding of neural circuits involved in punishment-resistant alcohol use not only in male but also in female subjects.

### Contribution of NAcSh to alcohol use despite negative consequences in females

We first found that lower activity in NAcSh was associated with lower punishment-imposed suppression of alcohol intake. In contrast to our previous study [27], we did not include a home-cage control group for baseline cFos expression. Such a group helps to identify if certain brain regions have differential activity for all types of training (punished or unpunished context). In addition, a yoked-shock control may have helped dissociate potential shock-induced cFos induction from punishment effects. Here we chose to make comparisons strictly between punished and unpunished conditions to reveal differences between these two. We argue this is the most important comparison for identifying brain regions involved in persistent alcohol consumption despite negative consequences. It remains possible that the shock experience in the punishment-resistant group contributes to the pattern of activity we observed, although by the final test session the rats with lower suppression ratio may have habituated to the expected shock outcome. Finally, we were statistically underpowered to determine whether the observed activity patterns were specific to male or female rats. While we found more male rats in the punishment-sensitive condition than female rats, we cannot disentangle whether activity patterns are specifically related to punishment resistance or sex.

We next used chemogenetic inhibition of NAcSh to test for a causal role in punishment-resistant alcohol consumption. Due to the population sex difference we observed, we increased the sample size and conducted separate analyses by sex to better explore the potential sex-dependent effects of NAcSh inhibition on punished alcohol intake. In previous studies, inhibition of NAcSh caused enhanced food consumption [65–68], or alcohol seeking in extinction [69, 70]. However, in our study, inhibition of NAcSh resulted exclusively in an effect on punished alcohol use, with no effect of NAcSh inhibition on unpunished alcohol intake, or in motivation for alcohol reward. At the higher shock intensity, NacSh inhibition exclusively increased alcohol intake in female rats with a higher punishment-resistance score. Thus, while our study indicates that there may be sex differences in the neurobiology of punished alcohol use, underrepresentation of punishment-resistant male rats in this study may also account for the lack of effect in males.

NAcSh is a functionally heterogenous brain region both in terms of cell type and anatomical boundaries. Different populations of NAcSh neurons along the dorsal-ventral axis encode motivationally opposing states [71], and others have shown distinct encoding of motivational states in NAcSh across the medio-lateral axis [72], as well as in the anterior-posterior axis [73, 74]. Perhaps most relevant to this study, is that identical NAcSh manipulations produce variable effects depending on the test conditions. For example, NAcSh inactivation causes aversive motivational states when tested in a stressful environment, but appetitive motivational states when tested in the home environment [75]. Thus, from this perspective of nucleus accumbens function, we propose that punishment-sensitive and -resistant rats display differential responses to the shock punishment, which may be reflected in distinct engagement of NAcSh.

## Conclusions

In this study we tested the role of NAcSh in unpunished and punished alcohol self-administration in male and female rats. We identified greater vulnerability in female rats to developing punishment-resistant alcohol consumption. Moreover, chemogenetic inhibition of NAcSh was found to exacerbate the punishment-resistant phenotype in female rats without affecting unpunished intake. This study raises the possibility that the neurobiology of punishment-resistance is sex-dependent. Gaining a deeper understanding of how NAcSh and associated neural circuits may be involved in alcohol use despite negative consequences in both sexes, could increase our understanding of the aetiology of AUD.

## Supplementary information

Supplementary Materials

## Data Availability

The datasets generated and analyzed from this study are available in the Open Science Framework (OSF) repository, https://osf.io/vnu46/.
