# Supplementary material for: Punishment-resistant alcohol intake is mediated by the nucleus accumbens shell in female rats
Source: Neuropsychopharmacology. 2024 Jul 29;49(13):2022–31. doi: 10.1038/s41386-024-01940-0 (PMC11480374; doi:10.1038/s41386-024-01940-0)
Supplement: Supplementary file 1 — Supplementary Materials [file 41386_2024_1940_MOESM1_ESM.docx]

**Supplemental Materials & Methods**

**Subjects**

During alcohol self-administration, mild food restriction was gradually put in place (males: 90%, females: 100% expected metabolic energy requirements) to prevent extreme weight gain previously seen in our male rats (>800 g) (Iossa et al., 1999; National Research Council (US) Subcommittee on Laboratory Animal Nutrition, 1995). Water was always available ad-libitum. See **Table S1** for housing details.

***Table S1.*** *Animal housing and husbandry details.*

| Cage | Same-sex pair-housed in conventional cages (Eurostandard type IV, Techniplast, Buguggiate, Italy).  Both sexes were housed in the same room. |
| --- | --- |
| Food | Teklad® Global 16% Protein Rodent Diet, Envigo, USA; Ad libidum access until the start of alcohol self-administration training, then rats were placed on mild food restriction (males: 90%, females: 100% expected metabolic energy requirements). Feeding took place daily after behavioural training, or at a similar time if training did not take place. |
| Water | Autoclaved water was always available ad libidum. |
| Bedding and nesting material | Spruce wood shavings were provided as bedding (Item #02132, Tecnilab-BMI, Someren, Netherlands). |
| Temperature/Humidity | Temperature (19-24°C) and humidity (55% ± 15%) were controlled daily |
| Sanitation (Cage change, water change, material transferred) | All materials (including water) were autoclaved before use. Cages were cleaned every other week (but kept enrichment material), and water bottles were replaced weekly. |
| Social environment | Rats were paired upon delivery and lived with the same cage mate throughout experiments. |
| Biosecurity level | Surgical facility: DMII |
| Lighting (type, schedule, intensity) | 12/12-hour reverse light/dark cycle (07h00 lights OFF). |
| Environmental enrichment | Cage enrichment included: nesting material, wood gnawing stick, and a red plexiglass tunnel shelter. |
| Sex of the experimenter | Both male and female experimenters worked with these cohorts, and rats were habituated by both genders before experiments began. |

**Viral Injection Surgical Procedure for chemogenetic experiments**

30-60 mins before surgery, rats were injected with analgesics carprofen (Rymadil®, 5 mg/kg, s.c.) and buprenorphine (Buprecare®, 0.01 mg/kg, s.c., Ecuphar NV, Belgium), to decrease inflammation and pain. Rats were anesthetized using isoflurane gas (PCH, Netherlands, 4% induction chamber, 2% maintenance through nose cone, Oxygen: 0.8 L/min, Pressurized Air: 0.3 L/min). Once anesthetized, rats were prepared for stereotaxic surgery under standard sterile conditions. After lidocaine (10 mg/kg, s.c.) was injected in the scalp, an incision was made, and the skull was leveled along the dorsal-ventral axis in the stereotaxic setup (David Kopf Instruments, USA). Craniotomies were made over injection coordinates (see below). Viral vectors were injected from a 10 μL Nanofil syringe (World Precision Instruments (WPI), USA), connected to micro syringe pump injector (UltraMicroPump, WPI) and SYS-Micro4 Controller (WPI) at a rate of 0.1 μL/min. After allowing 1 min/0.1μL diffusion time, needles were withdrawn, craniotomies were filled with bone wax, and the incision was stitched up. Rats were injected with 1 mL saline (NaCl, 0.9%, s.c.), to account for fluid loss. 24-48 hours post-surgery, rats were injected with carprofen (Rymadil®, 5 mg/kg, s.c.). Rats were monitored daily during the first week after surgery, and given extra care based on body weight and incision swelling. 7-10 days post-surgery, stitches were removed, and rats were transferred from the surgical unit back to the behavioural facility.

***Viral Vectors.*** For chemogenetic experiments, we used the following viral vectors, constructed by the University of Zurich Viral Vector Facility (UZH VVF, Zurich, Switzerland): a non-conditional chemogenetic hM4Di viral vector (**hM4Di,** AAV-5/2-hSyn1-hM4D(Gi)_mCherry-WPRE-hCHp(A), 6.3 x 10E12 vg/ml, UZH VVF, p107-5, Addgene viral prep #50475-AAV5) and an mCherry viral vector as a control (**mCherry**, AAV-5/2-hSyn1-chI-mCherry-WPRE-SV40p(A), 6.1 x 10E12 vg/ml, UZH VVF, p133-5, Addgene viral prep #14472-AAV5).

***NAcSh Inhibition Experiment.*** For NAcSh inhibition experiments, we bilaterally injected 0.5uL viral vector per hemisphere in NAcSh (AP+1.7, ML+/-2.4, DV-7.8 from Bregma) at a 10° angle.

***VTA/SNc inhibition experiment.*** In the VTA inhibition deschloroclozapine validation experiment, we bilaterally injected 0.5 uL viral vector per hemisphere in the VTA/SNc (AP-5.3, ML+/-2.6, DV-7.55 from Bregma) at a 10° angle.

**Alcohol**

We prepared 20% (v/v) ethanol (VWR Chemicals, Amsterdam, Netherlands) in autoclaved tap water to use for the alcohol behavioural procedures.

**Behavioural Procedure**

*Home-cage intermittent alcohol access two-bottle choice procedure*

At least one week after arrival, rats were same-sex pair-housed in their home cages and we began the intermittent alcohol access two-bottle choice procedure (Simms et al., 2008; Wise, 1973), similar to procedures in our previous experiments (Marchant et al., 2016, 2023; McDonald et al., 2021). Rats received 24-hour alcohol access (3 x per week) then 24-48 hours without access for 4 weeks, for a total of 12 x 24hr sessions.

During alcohol access, two bottles were placed on the home cage; one contained water and one contained 20% (v/v) ethanol. Bottles were removed after 24 hours and replaced with a regular water bottle. Bottles were then weighed to calculate alcohol intake and preference over water. The location of the alcohol bottle was switched every session to avoid side preference. We placed two additional bottles on empty cages to estimate spillage. Average spillage (g) was subtracted before calculating alcohol preference (%) and alcohol intake for each session. Body weight was measured twice per week and averaged to estimate alcohol intake in g/kg.

*Punished alcohol self-administration training procedure.*

After an acclimatization period (7-10 days), rats began alcohol self-administration in operant chambers (see **Apparatus**). Training took place during the dark phase, 5-6 days per week. Both sexes were trained at the same time, but specific operant chambers were designated as male- or female-only. Body weight was measured twice per week and averaged to estimate alcohol intake (g/kg).

**Phase 1: Magazine Training.** Rats first underwent 2 to 4 30-minute magazine training sessions. In this phase, every 150 seconds, a cue light and tone turned on for 3 seconds, and 0.08 mL alcohol was non-contingently delivered to the magazine. Magazine entries and alcohol intake were recorded. Alcohol intake (mL) was calculated as the number of alcohol deliveries subtracted by any alcohol leftover at the end of the session. Alcohol intake was then converted to g/kg accounting for each rat’s body weight.

**Phase 2: Alcohol Self-Administration.** To start the 30-minute sessions, levers extended, and the house light signalled alcohol availability. Rats learned to press the active lever to receive 0.12 mL 20% alcohol delivered to the magazine. Upon active lever press, the cue light and tone turned on for 3 seconds. If either lever was pressed during this 3-second period, presses were recorded but had no consequence. Inactive lever presses were recorded but had no consequence.

Alcohol self-administration sessions began at a fixed-ratio schedule of 1 (FR1), where each active lever press resulted in one alcohol reward delivery. Next, the requirement was gradually increased up to FR3, where 3 lever presses were required to obtain one alcohol delivery. In total, rats completed 25-35 alcohol self-administration sessions. Lever presses (active and inactive), alcohol deliveries and alcohol intake (g/kg) were recorded.

**Phase 3: Progressive Ratio Test.** Next, rats underwent progressive ratio test sessions to assess motivation to obtain alcohol (Richardson & Roberts, 1996).The progressive ratio session began at FR1, then after each alcohol delivery was obtained, FR was progressively increased by 1 (i.e. FR1, 2, 3, 4, 5, ...). The n+1 progressive ratio schedule was selected based on alcohol’s reward value, comparable to previous research (Randall et al., 2017), although other schedules have also been used (Domi et al., 2021; Giuliano et al., 2018). Sessions ended after 60 minutes, or after 30 minutes without reward. Additional behavioural outcome measures recorded in progressive ratio test sessions included breakpoint (the highest achieved FR).

**Alcohol Self-Administration Baseline:** After progressive ratio sessions, rats completed 3 to 5 final FR3 alcohol self-administration sessions, to use as a baseline before punished alcohol sessions began.

**Phase 4: Punished Alcohol Self-Administration.** During the punishment phase, sessions were identical to previous alcohol self-administration sessions, except now with a 33% probability of footshock upon the second active lever press in the FR3 sequence. We used a random selection function (RANND) from the following array (0,0,1) to determine whether the second response is to be punished. The resulting probability is that one out of every three of the second lever presses in the FR3 sequence is punished. Footshocks did not occur on the third active lever press in the FR3 sequence to avoid the possibility of counterconditioning if the alcohol and shock outcomes co-occurred (Marchant et al., 2018a; Randall et al., 2017). During the first punished alcohol sessions, shock intensity was set to 0.20 mA. After 3-5 punished alcohol self-admin sessions at the same intensity, the shock was increased. Sessions were run at 0.20 mA, 0.25 mA and 0.30 mA. In total, rats received 9 punished alcohol self-administration sessions. Additional behavioural outcome measures recorded during punished alcohol self-administration included the number of shocks received. A suppression ratio was also calculated (see below).

*Non-contingent footshock sensitivity procedure*

We ran a non-contingent footshock sensitivity test to confirm that all individuals (both males and females as well as punishment-sensitive and punishment-resistant alcohol responders) could sense and responded similarly to foot-shocks at the intensities we used in the punished alcohol self-administration procedure. To that effect, a separate cohort of rats (n = 24, 12 male, 12 female), underwent a foot-shock sensitivity test after the unpunished alcohol self-administration procedure.

We used an operant chamber unfamiliar to these rats. The metal grid floor was connected to an aversive stimulator/scrambler module (Item #ENV-414, Med Associates Inc.) to deliver foot-shocks. A Raspberry Pi night vision infrared camera (run from a 3 Model B+ Raspberry Pi computer) was mounted on the chamber door, to record a side view of the entire operant chamber and connected to an external monitor outside the chamber for real-time viewing. Videos were recorded (10 fps, 820x616 resolution) throughout the test to observe behavioural responses to shocks. A stimulus light was placed in view, which was used to signal the duration of the shocks for video analysis.

Each rat was first placed in the chamber and waited 5 minutes to begin the test. The test consisted of a total of 7 non-contingent shocks, starting at 0.05 mA, and increasing by 0.05 mA increments until 0.35 mA, each 0.5 s in duration. Each shock was only imparted if the rat’s 4 paws were touching the metal grid, which we viewed on the video monitor. We waited at least 1 minute until sending the next shock.

Offline, the behavioural response to each shock was manually analyzed from the recorded videos. Behavioural response during each shock was scored using the following scale: 0 – no effect, 1 – head orientation towards grid, 2 – paw lifted from metal grid, 3 – startle jump, 4 – escape jump (adapted from Golden et al., 2017).

**Perfusion, Brain Sectioning, Staining & Mounting**

Rats were first anesthetized with isoflurane gas (4% induction chamber Oxygen: 0.8 L/min, Pressurized Air: 0.3 L/min, PCH, Netherlands) and then given a lethal dose of pentobarbital (0.8 mL i.p., Euthasol®, AST Farma, Netherlands). Once breathing significantly slowed, and no paw reflex was observed, the rat was transcardially perfused. Before inserting the syringe pump, the heart was injected with 0.7 mL solution containing 1% sodium nitrite and heparin (5000 i.u./ml, Serva, Germany) to dilate blood vessels. Next, the rat was transcardially perfused first with saline (100 mL, 0.9%) and then paraformaldehyde (350-400 mL, 4% in 0.1 M sodium phosphate at pH 7.4) to fix the tissue. After perfusion, the brain was extracted and placed in 4% paraformaldehyde for ~2 hours to post-fix. Then, brains were transferred to 30% sucrose in phosphate-buffered saline (PBS). Brains were prepared for cryo-slicing with Tissue Tek (Sakura Finetek, Netherlands) and fast frozen in dry ice. Brains were then transferred to the cryostat (Thermo Fisher Scientific, USA) kept at -19 °C to -21 °C for slicing. We collected 4 series of 40 um coronal sections, stored in PBS at 4 °C until immunohistochemical staining and mounting could be performed.

*cFos Experiment (Experiment 2)*

***Staining.*** We performed immunohistochemical staining for cFos, an immediate-early gene protein product to identify activated neurons, using the avidin-biotin complex (ABC) DAB (diaminobenzidine) technique (Vectastain Elite ABC-HRP kit with peroxidase, Vector Laboratories, #PK-6100), similar to previous work (Marchant et al., 2016). After free-floating sections were washed in tris-buffered saline (TBS, 3 x 10 min washes), they were transferred to 0.3% H_2_O_2_ in TBS for 30 mins. After washed with 0.5% Triton X-100 in TBS (TBS-T), TBS and then TBS-T for 10 mins each. They were then placed in the blocking solution, 3% Normal Goat Serum (NGS) in TBS-T for 1 hour. Next, sections were incubated with the cFos rabbit primary antibody (1:2000, Phospho-c-Fos (Ser32(D82C12)**,** Cell Signaling Technology, USA) in 3% NGS in TBS-T for 48 hrs at 4 °C.

After the primary incubation period, sections were washed (3 x 10 min in TBS-T, TBS and TBS-T), and then transferred to the secondary antibody solution. Sections were incubated with Biotin goat-anti-rabbit secondary antibody (1:400 in 3% NGS in TBS-T, Jackson, UK, RRID: AB_2337965) for 2 hours. After secondary incubation, sections were washed (3 x 10 min in TBS). Then, sections were incubated in the ABC elite solution(1:400, Vectastain Elite ABC-HRP kit) in TBS-T for 1 hr. After sections were washed (3 x 10 min in TBS, TBS and Tris-HCl), they were incubated in DAB-Ni solution (diaminobenzidine, nickelammoniumsulfate, ammoniumchloride, glucose oxidase, D-glucose in Tris-HCl) for 11 minutes, and then washed (3 x 10 min in Tris-HCl). Stained sections were stored in PBS at 4 °C covered until mounting.

***Slide Mounting*.** We mounted sections onto slides (Thermo Scientific, USA) using 0.3% gelatin (Merck, Germany) in Tris-HCl solution, warmed on a hotplate (35 °C). After drying overnight, tissue was dehydrated through gradually increasing ethanol concentrations and xylene, 2 minutes per step (70, 80, 96, 96, 100, 100% ethanol, 3 x xylene) . Slides were cover-slipped (Marienfeld Superior, Germany) using Entellan (Sigma-Aldrich).

***Histological Image Acquisition.*** We digitally captured bright-field images of DAB-stained cFos-positive cells using a brightfield microscope (Leica) at 5x magnification.

***cFos-positive cell quantification*.** Slides were visualized using Fiji ImageJ software. Brain regions of interest were drawn, and cells were counted using the automatic analyze particles function in ImageJ. We analyzed the following brain regions: Bregma +4.68 mm: OFC, orbitofrontal cortex. Bregma +2.52 mm: PrL, prelimbic cortex; IL, infralimbic cortex; aIC, anterior insula. Bregma +2.04 mm: NAcC, nucleus accumbens core; NAcSh, nucleus accumbens shell. Anatomical boundaries of these regions were determined by the Rat Brain Atlas (Paxinos & Watson, 2005), and the representative images in Figure 2C show an approximately 1mm square sub-region of the region of interest that was analysed. Cell counts in each region were averaged across two sections per rat. cFos-positive cell counts were normalized to the area of the anatomical boundary that was measured by dividing the total count of positive cells by the area in mm, resulting in the data being presented as cFos/mm2.

*Chemogenetic Experiments (Experiments 2 & 3)*

***Staining.*** For chemogenetic experiments, viral-expressing cells also expressed mCherry for histological verification. We stained for DAPI (1:5000 in PBS, Sigma-Aldrich, USA) to mark cell nuclei. Sections were first washed in PBS (3 x 10 min washes), then kept in DAPI solution for 7 minutes, and then washed in PBS (3 x 10 min washes). Stained sections were stored in PBS at 4 °C until mounting.

***Slide Mounting***. We mounted sections onto slides (Thermo Scientific, USA) using 0.2% gelatin (Merck, Germany) in Tris-HCl solution, warmed on a hotplate (35 °C). Slides were cover-slipped (Marienfeld Superior, Germany) using a Mowiol mounting medium with DABCO (1,4-diazabicyclo-[2,2,2]-octane) to reduce fluorescence fading.

***Histological Image Acquisition.*** Slides were scanned using the Vectra Polaris slide scanner (Akoya Biosciences, USA), at 20 x magnification to image DAPI-stained cell nuclei (DAPI filter) and mCherry viral vector expression (Texas Red filter).

***Viral Expression Verification***. Slides were visualized using QuPath software version 0.3.0 (Bankhead et al., 2017). To verify hM4Di-mCherry expression in both chemogenetic experiments, viral expression was qualitatively analyzed by 2 different observers, blinded to virus group. Rats were excluded from chemogenetic test analyses if they did not show bilateral viral expression around the injection coordinates.

**Experimental Design**

*Experiment 3. Novel chemogenetic ligand deschloroclozapine behavioural validation*

A separate cohort of 8 rats (4 male, 4 female) underwent surgery to inject either hM4Di or mCherry viral vectors in the VTA/SNc (**Figure S4A**). After at least 4 weeks, we conducted open field tests to identify the behavioural effects inhibiting VTA/SNc with the novel chemogenetic ligand, deschloroclozapine (DCZ) (Nagai et al., 2020), in rats. Tests were completed in a within-subject repeated-measures design, where each rat received saline (SAL), 0.05 mg/kg DCZ dose, and 0.10 mg/kg DCZ dose on separate days (Nagai et al., 2020; Nentwig et al., 2021). After ligand injection, each rat was placed in a dark open field arena (73.5 x 51 x 43 cm) for 30 minutes. Total distance travelled (cm) was calculated from recorded videos using ezTrack, an open-source tracking software (Pennington et al., 2019).

**Statistical Analyses**

Normality was checked using the Shapiro-Wilk test and Q-Q plot visualization. If statistical assumptions were not approximately met, non-parametric alternatives were used.

*Experiment 1. Behavioural characterization of punished alcohol self-administration in male and female rats*

We used separate two-way repeated-measures ANOVAs to analyze most behavioural variables from punished alcohol self-admin phases across Session (within-subject factor) and between Sex or Punishment-Resistance (between-subject factors). For the home-cage phase, behavioural variables included: alcohol intake (g/kg) and alcohol preference (%). For the unpunished alcohol self-admin phase: total active lever presses and alcohol intake (g/kg). Additionally for the punished alcohol self-admin phase: Suppression Ratio (SR) and footshocks. For the progressive ratio test, we used separate unpaired t-tests to analyze total active lever presses and breakpoint. To identify the effect of punishment on alcohol self-administration, we also analyzed SR using individual one-sample Wilcox tests. Finally, a Chi-squared test of independence was conducted to identify differences in the number of punishment-resistant male and female rats.

*Experiment 2. Neuronal activity associated with punished alcohol self-administration.*

We used separate one-way ANOVAs to analyze total active lever presses and alcohol intake (g/kg) with Test Group (Unpunished, Punishment-Sensitive, Punishment-Resistant) as a between-subject factor. For rats who completed the punished test, we used separate unpaired t-tests to analyze SR and footshocks with Punishment-Resistance as a between-subject factor. We used separate Spearman’s correlations to assess the relationship between cFos neuronal activity and test behaviour (total active lever presses, alcohol intake (g/kg), SR). Finally, we analyzed cFos total cell counts in each brain region separately using one-way ANOVAs with Test Group as a between-subject factor.

*Experiment 3. Novel chemogenetic ligand deschloroclozapine behavioural validation*

Repeated measures ANOVAs were conducted on total distance travelled (cm), with Virus (mCherry, hM4Di) as a between-subject factor, and Ligand (Saline, 0.05 mg/kg DCZ, 0.10 mg/kg DCZ) and time bin (0-30 minutes, 5-minute bins) as within-subject factors.

*Experiment 4. NAcSh chemogenetic inhibition during punished alcohol self-administration*

We used generalized linear mixed models (GLMM) (Bolker et al., 2009) with the glmmTMB R package (Brooks et al., 2017) to identify Virus x Ligand x Punishment-Resistance interaction effects on test behaviour separately for each sex. Using GLMMs allowed us to preserve Punishment-Resistance score as a continuous variable and therefore helped us identify potential differential effects of NAcSh inhibition between punishment-sensitive and punishment-resistant individuals. We could also account for the non-normal distributions of the dependent variables. Total active lever presses (positive integers with many zeroes) could be modeled by a negative binomial distribution (Dunn & Smyth, 2018). Alcohol intake (g/kg) and Suppression Ratios (SR) (positive continuous data with many zeroes) were best approximated by a Tweedie distribution (1 < ξ < 2) (Dunn & Smyth, 2018). Model predictions were plotted from synthetic data to better visualize significant Virus x Ligand x Punishment-Resistance interactions effects.

**Supplemental Results**

***Experiment 1. Behavioural characterization of punished alcohol self-administration in male and female rats***

*More female rats become punishment-resistant after higher alcohol self-administration rates.*

***Home-cage alcohol intake.*** **Figure 1A** shows the behavioural procedure. During the home-cage alcohol access phase, we found a main effect of Session (*F*(5.32,159.68)=17.19, *p*<.001, η^2^_G_=0.17) on alcohol intake (g/kg) showing increased alcohol consumption from the first to the last session (*p*<.001) (**Figure 1E).** We did not observe a significant effect of Sex (*F*(1,30)=0.29, *p*=.597), nor a Sex x Session interaction (*F*(5.32,159.68)=0.96, *p*=.450). For alcohol preference (%) over water (**Figure S1)**, we did find a significant Sex x Session interaction (*F*(3.6,107.89)=2.77, *p*=.036, η^2^_G_=0.04), which revealed that Males had higher preference for alcohol than Females during the last 4 sessions (*Session 9: p=.018, 10: p=.011, 11: p=.001, 12: p=.022*). Male rats showed a significant increase in alcohol preference from the first to the last session (*p*=.004), but female rats did not (*p*=.364).

***Alcohol Self-Administration.*** In alcohol self-administration, we examined active lever pressing and alcohol intake (g/kg) from the final 3-day average of each fixed ratio (FR1, FR2, FR3) (**Figure 1F**). We found a significant Sex x Session interaction (*F*(1.53,91.98)=6.88, *p*=.004, η^2^_G_=0.06) in active lever pressing, which revealed that Males showed higher lever pressing at FR1 (*p*=.026), but Female rats showed higher lever pressing rates by FR3 (*p*=.016). There was also a main effect of Session in both Male (*p*=.006) and Female (*p*<.001) rats. Female rats increased lever pressing from FR1 to FR2 (*p*=.002) and FR2 to FR3 (*p*<.001), while Male rats only significantly increased lever pressing from FR2 to FR3 (*p*=.036).

We conducted a separate analysis for alcohol intake (g/kg) (**Figure 1F**), and similarly observed a significant Sex x Session interaction (*F*(2,120)=4.63, *p*=.012, η^2^_G_=0.04), which showed that Female rats had higher alcohol intake than Males for FR2 (*p*=.005) and FR3 (*p*<.001). We also found an effect of Session in Male rats (*p*<.001) but not Female rats (*p*=.093), in which Male rats consumed significantly less alcohol in FR2 versus FR1 (*p*<.001) but maintained consumption levels from FR2 to FR3 (*p*=.372).

***Progressive Ratio Test.*** Total active lever presses and the breakpoint, the final completed fixed ratio, from the progressive ratio test are shown in **Figure 1G**. We found that Female rats showed higher active lever presses compared to Males (*W*=292, *p*=.011). Similarly, there was a significant effect of Sex on breakpoint (T-test, *t*(60)=-2.78, *p*=.007), in which Female rats had higher breakpoints than Males.

***Punished Alcohol Self-Admin.*** See **Figure 1A,C,D** for the punished alcohol self-administration procedure. Overall, punishment significantly decreased alcohol self-administration from baseline (SR=0.5) for every session after the first session (individual one-sample Wilcox tests, (P1: W=688, *p*=.099; P2-P9: *Ws<*216, *ps*<.001 (**Figure 1H**). We found comparable results in both Male rats (P1: *W*=130, *p*=.078; P2-P9: *Ws*<21, *ps*<.001) and Female rats (P1: *W=*234, *p*=.399; P2-P9: *Ws*<101, *ps*<.001). Using individual suppression ratios from each session, we did not find a significant Sex x Session interaction (*F*(6,359.73)=1.79, *p*=.10), but we did observe main effects of Sex (*F*(1,60)=9.25, *p=*.003, η^2^_G_=0.06) and Session (*F*(6,359.73)=62.95, *p*<.001, η^2^_G_=0.37) separately (**Figure 1H**). Female rats showed higher SRs compared to Males in sessions P3 (*p*<.001), P4 (*p*=.03), P7 (*p*=.006) and P8 (*p*=.009). We also found an effect of Session in both Male and Female rats (*ps*<.001) in which Male rats significantly decreased SR from P1 to P2 (*p=*.015) and Female rats significantly decrease SR from both P1 to P2 (*p*=.013) and P4 to P5 (*p=*0.024), but no other sessions.

We calculated a Punishment-Resistance score from the average SR from the final two punishment sessions at 0.25 mA (**Figure 1I**). The top 33% of rats were classified as “Punishment-Resistant” (PR, *n*=22), while the bottom 33% were classified as “Punishment-Sensitive” (PS, *n*=20) (**Figure 1I**). Using a Chi-square test of independence, we observed that the relationship between Sex and Punishment-Resistance was significant (*X^2^*(2)=6.217, *p*=.045), indicating that fewer males and more females than expected were classified as Punishment-Resistant (**Figure 1I, Table S2**).

***Table S2.*** *Punishment-Resistance category by Sex. Count, expected values, Chi-square contribution, Row%, Column%, Total%.*

|  | Pun-Sensitive | Mid | Pun-Resistant | Total |
| --- | --- | --- | --- | --- |
| Male | 13  8.7  2.11  48.2%  65.0%  21.0% | 8  8.7  0.06  29.6%  40.0%  13.0% | 6  9.6  1.34  22.2%  27.0%  9.7% | 27  43.6% |
| Female | 7  11.3  1.6  20.0%  35.0%  11.3% | 12  11.3  0.05  34.3%  60.0%  19.4% | 16  12.4  1.0  45.7%  72.7%  25.8% | 35  56.4% |
| Total | 20  32.3% | 20  32.3% | 22  35.5% | 62  100% |

*Punishment-resistance emerges without prior differences in unpunished alcohol intake*

See **Figure 1A,C** for punished alcohol self-administration procedure details. Overall, punishment significantly decreased alcohol self-administration from baseline (SR=0.5) for every session except the first (individual one-sample Wilcox tests, (P1: W=688, *p*=.099; P2-P9: *Ws<*216, *ps* <.001 (**Figure 1H**). Consistent with past work, some individuals continued alcohol intake at a similar rate to unpunished sessions (SR =~0.5), while others greatly decrease their responding (SR<0.2) (**Figure 1H**). We calculated the average Suppression Ratio (SR) from the final two punishment sessions at 0.25 mA as the Punishment-Resistance score (**Figure 1I**). We selected this score because we saw large individual differences in alcohol intake due to punishment, and the 0.25 mA shock intensity reliably produced similar behavioural response (small jump) across all individuals (**Figure S3)**.

***Active Lever Pressing and Alcohol Intake.*** Similar to results from active lever pressing, we found a significant Punishment-Resistance Score x Session interaction (*F*(4.53,181.18)=4.97, *p*<.001, η^2^_G_=0.05) when accounting for alcohol intake in g/kg (**Figure 1L**). Punishment-resistant and -sensitive rats showed similar alcohol intake at baseline (*p*=.737) and the first punished session (P1, *p=*.958), but punishment-resistant drank more during all other punished sessions versus punishment-sensitive (P2: *p*=.007, P3: *p*=.004; P4-P8: *ps*<.001; P9: *p=*.004), not only in the sessions from which they were classified (P4-P5) (**Figure 1L**). There was a significant effect of Session in both punishment-resistant and punishment-sensitive groups (*ps*<.001). Punishment-resistant rats significantly decreased alcohol intake from the first to the second 0.25 mA session (P4-P5, *p*=.014) while punishment-sensitive rats only significantly decreased alcohol intake from the first to the second session at 0.20 mA (P1-P2, *p*=.033).

***Experiment 2. Neuronal activity associated with punished alcohol self-administration.***

***Test Behaviour***. **Figure 2B** shows behavioural measures from the cFos test. We identified a Test Group effect for total active lever presses (*F*(2,8.43)=17.0, *p*=.001, η^2^_G_=0.48) in which punishment-sensitive rats completed less lever presses than punishment-resistant rats (*p=.003*) or unpunished rats (*p=.005*). Unpunished and punishment-resistant rats completed a similar amount of lever presses (*p=.991*). For alcohol intake (g/kg), we also found a Test Group effect (*H*(2)=12.8, *p*=.002, η^2^_H_=0.51) where punishment-sensitive rats consumed less alcohol than unpunished (*p*=.051) or punishment-resistant rats (*p*=.001). Punishment-resistant and unpunished rats consumed similar amounts of alcohol (*p*=.374). Suppression Ratio reflected differences in alcohol self-administration during the final punishment test, (*t*(16)=-6.98, *p*<.001) and we found that rats classified as punishment-resistant received more shocks than punishment-sensitive (Wilcoxon rank sum test, *W*=8.0, *p*=.003).

***Table S3.*** *Correlations between total cFos positive cells and test behaviour.* *(r_s_ and p-values)*

| Brain Region | Active Lever Presses | | Alcohol intake (g/kg) | | Suppression Ratio | |
| --- | --- | --- | --- | --- | --- | --- |
|  | Unpunished | Punished | Unpunished | Punished | Unpunished | Punished |
| OFC | -0.14  .80 | -0.44  .10 | -0.14  .78 | -0.45  .092 | --- | -0.39  .15 |
| PrL | -0.43  .42 | 0.10  .72 | -0.41  .42 | 0.04  .88 | --- | 0.09  .75 |
| IL | 0.1  .80 | -0.16  .57 | -0.20  .70 | -0.23  .41 | --- | -0.18  .53 |
| aIC | -0.43  .42 | -0.31  .24 | -0.64  .17 | -0.33  .22 | --- | -0.29  .28 |
| NAcC | -0.31  .56 | **-0.60***  **.014** | -0.06  .91 | **-0.55***  **.027** | --- | **-0.57***  **.022** |
| NAcSh | -0.37  .50 | **-0.60***  **.018** | -0.67  .15 | **-0.63***  **.012** | --- | **-0.59***  **.020** |

*Orbitofrontal Cortex, PrL = Prelimbic Cortex, IL = Infralimbic Cortex, aIC = Anterior Insula, NAcC = Nucleus Accumbens Core, NAcSh = Nucleus Accumbens Shell. *p < .05*

***Experiment 3. Novel chemogenetic ligand deschloroclozapine behavioural validation***

To test the effectiveness of the chemogenetic receptor ligand deschloroclozapine (DCZ) (Nagai et al., 2020) in rats, we tested different DCZ doses (0.05 mg/kg and 0.10 mg/kg) on spontaneous locomotor behaviour in rats expressing the inhibitory hM4Di receptor VTA/SNc (**Figure S4**) (Marchant, Whitaker, et al., 2016). Rats were injected with hM4Di or mCherry in the VTA/SNc, and we subsequently measured distance traveled (cm) over 30 minutes in open field locomotion tests where rats received saline, 0.05 mg/kg DCZ or 0.10 mg/kg DCZ in a repeated-measures within-subject design (**Figure S4A,B**). All rats showed expression of hM4Di (*n* = 4 (2 male, 2 female)) or mCherry (*n* = 4 (2 male, 2 female)).

We found a significant Virus x Ligand x Time interaction (*F*(10,40)=2.76, *p*=.011, η^2^_G_=0.109) on total distanced travelled (cm/30 mins) (**Figure S4C)**. Post hoc two-way ANOVA revealed a significant Virus x Time interaction for both 0.05 mg/kg DCZ (*F*(5,25)=4.20, *p*=.007, η^2^_G_=0.296) and 0.10 mg/kg DCZ (*F*(5,25)=8.36, *p*<.001, η^2^_G_=0.386), but not for saline (*F*(5,30)=30.50, *p*=.400). For the 0.05 mg/kg DCZ dose, we found a decrease in locomotion in hM4Di rats only from 5-10 minutes (*p*=.006) but no other time bins in the 30-minute period. For the 0.10 mg/kg DCZ dose, hM4Di rats showed a significant decrease in distance travelled during all time bins from 0 to 25 minutes (0-5 min: *p*=.004, 5-10 min: *p*<.001, 10-15 min: *p*<.001, 15-20 min: *p*=.007, 20-25min: *p*=.003, 25-30min: *p*=.326). In summary, we have shown that chemogenetic inhibition of VTA/SNc using DCZ significantly lowers distance travelled in both the 0.05 mg/kg and 0.10 mg/kg doses. Since the 0.10 mg/kg DCZ dose showed a sustained effect across 30 mins, we used this dose for all further chemogenetic experiments.

***Table S4.*** *Effects of NAcSh Inhibition in males and females on active lever pressing, alcohol intake and suppression ratio in chemogenetic tests.*

| **Males** | | | | | | |
| --- | --- | --- | --- | --- | --- | --- |
|  | ***Unpunished*** | ***Progressive Ratio*** | | ***0.25mA Punished*** | | ***0.30mA Punished*** |
| **Active Lever Presses** | | | | | | |
| Virus-hM4Di x Ligand-DCZ | *β*=0.45, SE=0.71, *Z*(42)=0.64, *p*=.525 | *β*=-0.29, SE=0.66, *Z*(42)=-0.44, *p*=.661 | *β*=1.30, SE=0.97, *Z*(42)=1.33, *p*=.182 | | *β*=0.11, SE=1.34, *Z*(42)=0.082, *p*=.935 | |
| Virus-hM4Di x Ligand-DCZ x Punishment-Resistance Score | *β*=-0.82, SE=2.71, *Z*(42)=-0.30, *p*=.761 | *β*=1.26, SE=2.55, *Z*(42)=0.50, *p*=.620 | *β*=-6.13, SE=3.53, *Z*(42)=-1.74, *p*=.083 | | *β*=-0.67, SE=4.80, *Z*(42)=-0.14, *p*=.889 | |
| **Alcohol Intake (g/kg)** | | | | | | |
| Virus-hM4Di x Ligand-DCZ | *β*=0.21, SE=0.61, *Z*(42)=0.34, *p*=.732 | *β*=-0.10, SE=0.43, *Z*(42)=-0.24, *p*=.809 | *β*=1.96, SE=1.39, *Z*(42)=1.40, *p*=.160 | | *β*=2.43, SE=3.01, *Z*(42)=0.81, *p*=.419 | |
| Virus-hM4Di x Ligand-DCZ x Punishment-Resistance Score | *β*=-0.47, SE=2.30, *Z*(42)=-0.20, *p*=.839 | *β*=0.36, SE=1.65, *Z*(42)=0.22, *p*=.828 | *β*=-8.40, SE=4.99, *Z*(42)=-1.68, *p*=.093 | | *β*=-5.52, SE=9.08, *Z*(367)=-0.61, *p*=.543 | |
| **Suppression Ratio** | | | | | | |
| Virus-hM4Di x Ligand-DCZ | *---* | *---* | *β*=1.46, SE=0.93, *Z*(50)=1.58, *p*=.115 | | *β*=0.01, SE=1.39, *Z*(42)=0.004, *p*=.997 | |
| Virus-hM4Di x Ligand-DCZ x Punishment-Resistance Score | *---* | *---* | *β*=-5.71, SE=3.21, *Z*(42)=-1.78, *p*=.076 | | *β*=-0.57, SE=4.88, *Z*(42)=-0.12, *p*=.908 | |
| **Females** | | | | | | |
| **Test** | ***Unpunished*** | ***Progressive Ratio*** | | ***0.25mA Punished*** | | ***0.30mA Punished*** |
| **Active Lever Presses** | | | | | | |
| Virus-hM4Di x Ligand-DCZ | *β*=0.70, SE=0.63, *Z*(50)=1.12, *p*=.262 | *β*=0.70, SE=0.63, *Z*(50)=1.12, *p*=.262 | *β*=1.53, SE=1.02, *Z*(50)=1.51, *p*=.132 | | *β*=-0.73, SE=1.76, *Z*(50)=-0.41, *p*=.679 | |
| Virus-hM4Di x Ligand-DCZ x Punishment-Resistance Score | *β*=0.86, SE=2.27, *Z*(50)=0.38, *p*=.710 | *β*=-1.18, SE=1.90, *Z*(50)=-0.62, *p*=.534 | *β*=-5.14, SE=2.82, *Z*(50)=-1.82, *p*=.069 | | *β*=4.59, SE=4.50, *Z*(50)=1.02, *p*=.308 | |
| **Alcohol Intake (g/kg)** | | | | | | |
| Virus-hM4Di x Ligand-DCZ | *β*=0.33, SE=0.65, *Z*(50)=0.51, *p*=.614 | *β*=0.64, SE=0.42, *Z*(50)=1.53, *p*=.126 | *β*=1.37, SE=1.15, *Z*(50)=1.23, *p*=.221 | | *β*=-3.72, SE=2.12, *Z*(50)=-1.75, *p*=.080 | |
| Virus-hM4Di x Ligand-DCZ x Punishment-Resistance Score | *β*=0.33, SE=2.01, *Z*(50)=0.16, *p*=.870 | *β*=-1.33, SE=1.24, *Z*(50)=-1.07, *p*=.286 | *β*=-4.50, SE=3.14, *Z*(50)=-1.44, *p*=.151 | | *β*=10.79, SE=5.37, *Z*(50)=2.01, ***p*=.045*** | |
| **Suppression Ratio** | | | | | | |
| Virus-hM4Di x Ligand-DCZ | *---* | *---* | *β*=0.78, SE=0.88, *Z*(50)=0.89, *p*=.375 | | *β*=-1.25, SE=1.48, *Z*(50)=-0.85, *p*=.398 | |
| Virus-hM4Di x Ligand-DCZ x Punishment-Resistance Score | *---* | *---* | *β*=-2.98, SE=2.50, *Z*(50)=-1.19, *p*=.234 | | *β*=4.94, SE=3.81, *Z*(50)=1.29, *p*=.196 | |

# **Supplemental Figures**


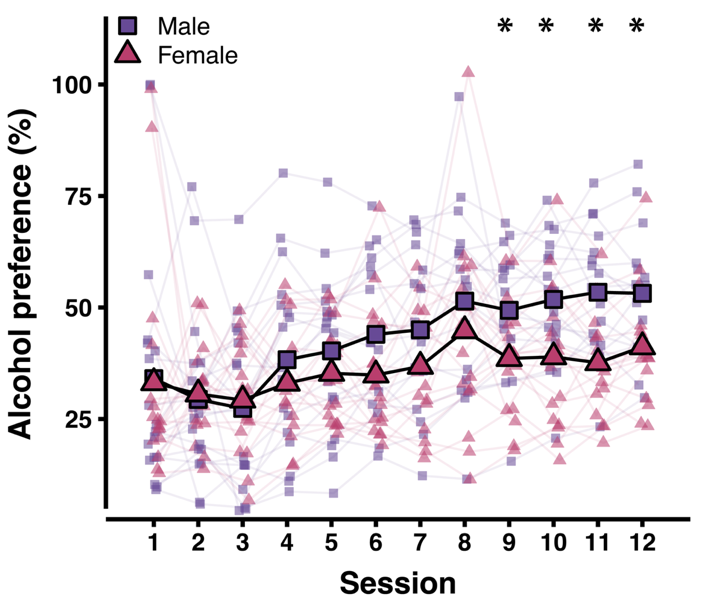


***Supplementary Figure S1.*** *Home-Cage Alcohol intake preference (%) versus water in males (purple squares) and females (pink triangles), across 12 sessions. *Difference between sexes, p<.05. Males show significantly higher preference for alcohol than females during the final four alcohol home-cage sessions, although this is not reflected in alcohol intake (g/kg) (****Figure 1E****), where there is no difference between male and female intake.*

**
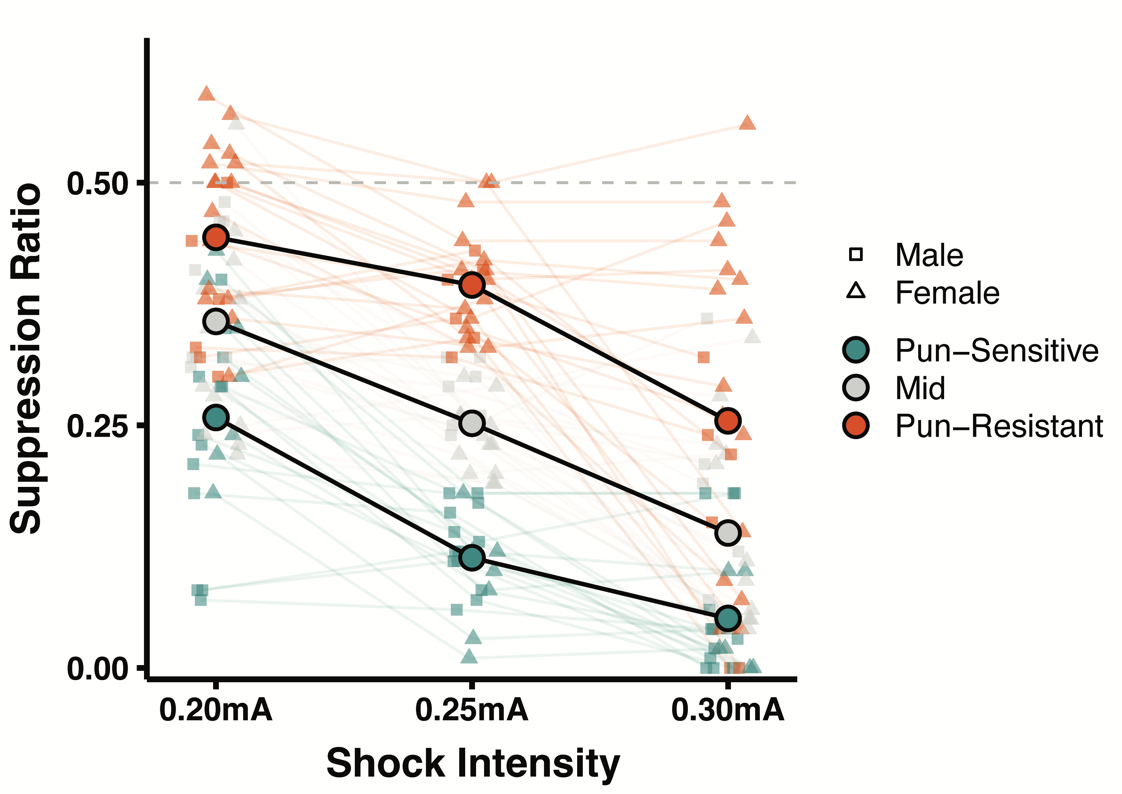
**

***Supplementary Figure S2****. Punishment-resistance score is stable across shock intensities at the extreme ends of each phenotype.* ***(Top)*** *Average suppression ratios across shock intensities (0.20mA-0.30mA). Rats with high Highest 33% SR at 0.25 mA are categorized as punishment-resistant (n=22, orange), middle 33% as middle (n = 21, grey) lowest 33% are punishment-sensitive (n=20, green). (Males = squares, females = triangles). (****Bottom****) Heat plot showing average suppression ratios across shock intensities (0.20mA-0.25mA) (PS: punishment-sensitive (SR <0.25), green; Mid, grey; PR: punishment-resistant (SR>=0.25), orange). Overall, the extreme ends of the phenotypes are stable. Rats who are punishment-resistant at 0.30mA are punishment-resistant at lower shock intensities. Rats who start as punishment-sensitive at 0.20mA stay punishment-sensitive at higher shock intensities. More rats start off as punishment-resistant at 0.20mA, and approximately 1/3 rats switch from punishment-resistant to punishment-sensitive with each 0.05mA shock increase.*

***Supplementary Figure S3****. Non-contingent footshock sensitivity test, analyzed by* ***(Left)*** *Punishment-Resistance Score (alcohol punishment-sensitive = green, alcohol punishment-resistant = orange) or* ***(Right)*** *Sex (male = purple, female = pink). Average behavioural response is plotted for increasing footshock intensities from 0.05 mA to 0.35 mA. 0 = no response, 1 = head orientation towards footshock grid, 2 = paw lifted from footshock grid, 3 = startle jump, 4 = escape jump. No differences in behavioural response to increasing non-contingent footshocks were observed between (left) punishment-resistant (n=5) and punishment-sensitive (n=14) alcohol responders or between (right) males (n=10) and females (n=9).*


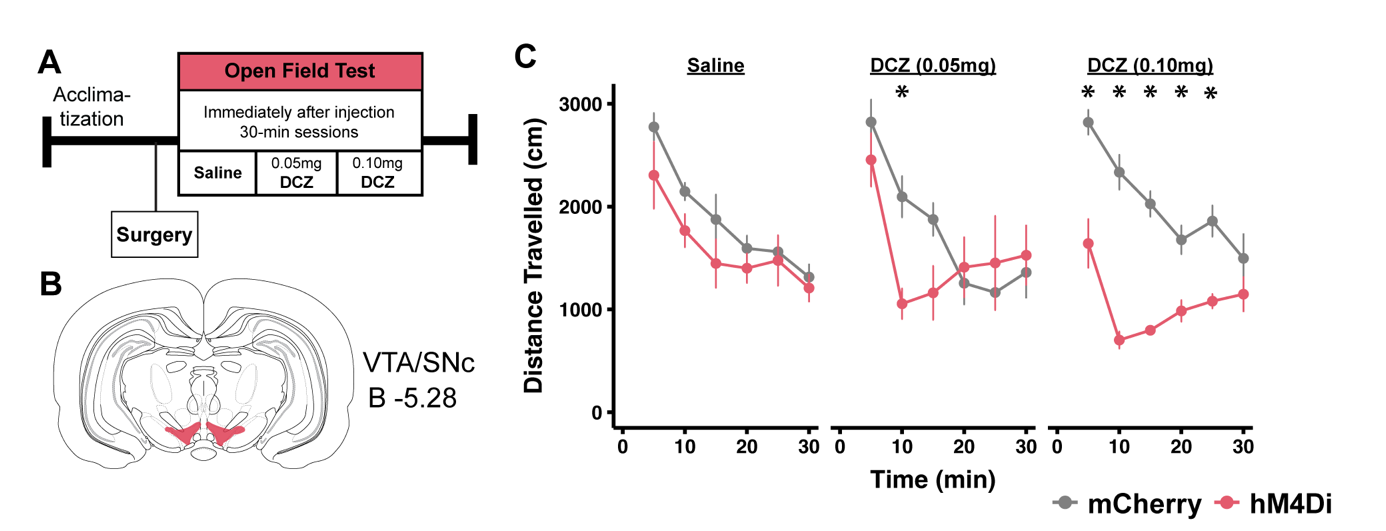
***Supplementary Figure S4.*** *Behavioural validation of the chemogenetic ligand deschloroclozapine (DCZ) by observing locomotor activity during VTA/SNc inhibition.* ***(A)*** *Experimental timeline.* ***(B)*** *Viral injection location of bilateral hM4Di-mCherry or mCherry control expression in VTA/SNc.* ***(C)*** *Total distance travelled (cm) in 30-minute open field tests expressed as Mean ± SEM of 5 min bins from rats expressing hM4Di (n = 4) or mCherry (n = 4), injected with Saline, 0.05 mg/kg DCZ, or 0.10 mg/kg DCZ. DCZ, deschloroclozapine. VTA, ventral tegmental area; SNc, substantia nigra. *Difference between viral group, p <.05.*

*
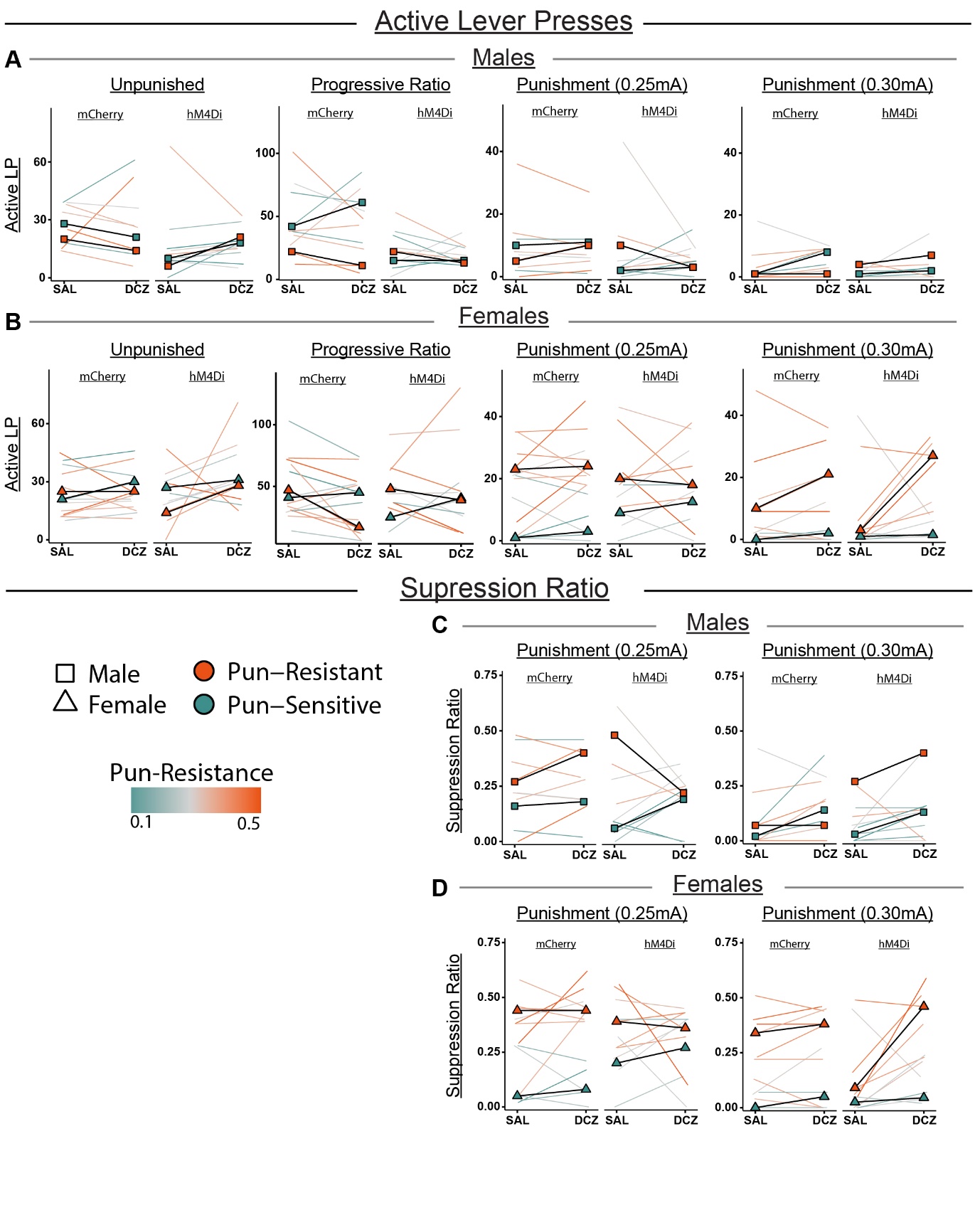
*

***Supplementary Figure S5.*** ***Chemogenetic inhibition of NAcSh during punished alcohol consumption****.* ***(A, B)*** *Effect of NAcSh inhibition on active lever presses in (A) males (mCherry: n = 10, hM4Di: n = 11) and (B) females (mCherry: n = 14, hM4Di: n = 11) during alcohol self-admin, progressive ratio test, punished alcohol self-administration at 0.25 mA and -punished alcohol self-administration at 0.30 mA.* ***(C, D)*** *Effect of NAcSh inhibition on suppression ratio in (C) males and (D) females during punished alcohol self-admin at 0.25 mA and -punished alcohol self-admin at 0.30 mA. Data expressed as Punishment-Resistance Score (Punishment-sensitive = green, punishment-resistant = orange) medians and individual datapoints. Individual datapoints are coloured using the continuous variable of Punishment-Resistance Score (Low = green, mid = grey, high = orange) and sex is indicated by squares (males) and triangles (females). Separate graphs in each panel show mCherry (left) or hM4Di rats (right) and each graph compares the repeated measure factor Ligand (Saline (SAL) vs. Deschloroclozapine (DCZ)).*
